# Performance of the Omnipod Personalized Model Predictive Control Algorithm with Meal Bolus Challenges in Adults with Type 1 Diabetes

**DOI:** 10.1089/dia.2018.0138

**Published:** 2018-09-01

**Authors:** Bruce A. Buckingham, Mark P. Christiansen, Gregory P. Forlenza, R. Paul Wadwa, Thomas A. Peyser, Joon Bok Lee, Jason O'Connor, Eyal Dassau, Lauren M. Huyett, Jennifer E. Layne, Trang T. Ly

**Affiliations:** ^1^ Division of Pediatric Endocrinology, Department of Pediatrics, Stanford University, Stanford, California.; ^2^ Diablo Clinical Research, Walnut Creek, California.; ^3^ Barbara Davis Center for Diabetes, University of Colorado School of Medicine, Aurora, Colorado.; ^4^ ModeAGC LLC, Palo Alto, California.; ^5^ Insulet Corporation, Billerica, Massachusetts.; ^6^ Harvard John A. Paulson School of Engineering and Applied Sciences, Harvard University, Cambridge, Massachusetts.

**Keywords:** Artificial pancreas, Automated insulin delivery, Closed-loop, Omnipod, tubeless Insulin pump, Postprandial

## Abstract

***Background:*** This study assessed the safety and performance of the Omnipod^®^ personalized model predictive control (MPC) algorithm using an investigational device in adults with type 1 diabetes in response to overestimated and missed meal boluses and extended boluses for high-fat meals.

***Materials and Methods:*** A supervised 54-h hybrid closed-loop (HCL) study was conducted in a hotel setting after a 7-day outpatient open-loop run-in phase. Adults aged 18–65 years with type 1 diabetes and HbA1c 6.0%–10.0% were eligible. Primary endpoints were percentage time in hypoglycemia <70 mg/dL and hyperglycemia ≥250 mg/dL. Glycemic responses for 4 h to a 130% overestimated bolus and a missed meal bolus were compared with a 100% bolus for identical meals, respectively. The 12-h postprandial responses to a high-fat meal were compared using either a standard or extended bolus.

***Results:*** Twelve subjects participated in the study, with (mean ± standard deviation): age 35.4 ± 14.1 years, diabetes duration 16.5 ± 9.3 years, HbA1c 7.7 ± 0.9%, and total daily dose 0.58 ± 0.19 U/kg. Outcomes for the 54-h HCL period were mean glucose 153 ± 15 mg/dL, percentage time <70 mg/dL [median (interquartile range)]: 0.0% (0.0–1.2%), 70–180 mg/dL: 76.1% ± 8.0%, and ≥250 mg/dL: 4.5% ± 3.6%. After both the 100% and 130% boluses, postprandial percentage time <70 mg/dL was 0.0% (0.0–0.0%) (*P* = 0.50). After the 100% and missed boluses, postprandial percentage time ≥250 mg/dL was 0.2% ± 0.6% and 10.3% ± 16.5%, respectively (*P* = 0.06). Postprandial percentages time ≥250 mg/dL and <70 mg/dL were similar with standard or extended boluses for a high-fat meal.

***Conclusions:*** The Omnipod personalized MPC algorithm performed well and was safe during day and night use in response to overestimated, missed, and extended meal boluses in adults with type 1 diabetes.

## Introduction

Artificial pancreas (AP) system development is an area of intense interest and rapid growth, with many patients, caregivers, and healthcare providers recognizing the potential of automated insulin delivery systems to improve glycemic outcomes and reduce the burden of diabetes care.^1–5^ Nearly every component of an AP system can be customized from a variety of available options, potentially leading to differences in system usability and performance.^6–8^ The algorithm driving insulin delivery (and glucagon for dual-hormone systems) for commercially available AP systems must be sufficiently robust to handle challenges to glycemic control that will be encountered as part of everyday use, while maintaining safety as the highest priority.^7,9^

First-generation hybrid closed-loop (HCL) systems will require the user to give meal boluses based on estimated meal carbohydrate (CHO) content and, as with current open-loop (OL) therapy, some boluses will be overestimated, underestimated, or missed.^10–12^ For example, Meade and Rushton reported that adults with diabetes scored an average of 59% on correctly estimating the CHO content of foods they ate frequently, with 82% overestimating CHO content by an average of 40%.^12^ This would result in an overestimation of the meal bolus amount and increase the risk of hypoglycemia. An overestimated bolus may also occur when the user delivers a bolus for but does not consume his or her entire meal.

There have been a limited number of studies examining the ability of single- and dual-hormone AP systems to safely respond to common real-life use cases of overestimated, underestimated, or missed meal boluses.^13–17^ These studies have generally shown that use of an AP system may help to avoid hypoglycemia after an overestimated bolus in most subjects by predictively reducing or suspending insulin delivery as needed based on the glycemic trajectory, which would not occur during standard OL therapy.^13,14^ Similarly, AP systems may reduce the severity and duration of hyperglycemia experienced after an underestimated or missed meal bolus compared with standard OL therapy by increasing insulin delivery in response to elevated glucose levels.^14–17^ In addition to functioning safely with overestimated or missed boluses, it may be advantageous for an AP system to provide flexibility to users by safely allowing extended boluses for high-fat meals,^18^ which has not been studied previously.

The Omnipod Horizon™ Automated Glucose Control System is a single-hormone HCL system using a personalized model predictive control (MPC) algorithm under development.^19,20^ Initial feasibility studies have demonstrated that the MPC algorithm performed well and was safe in adult, adolescent, and pediatric subjects with type 1 diabetes.^20^

The objective of this study was to evaluate the safety and performance of the Omnipod personalized MPC algorithm in adults with type 1 diabetes in a supervised outpatient hotel setting in response to overestimated and missed meal boluses, and extended boluses for high-fat meals.

## Materials and Methods

### Study design

**FIG. 1.**
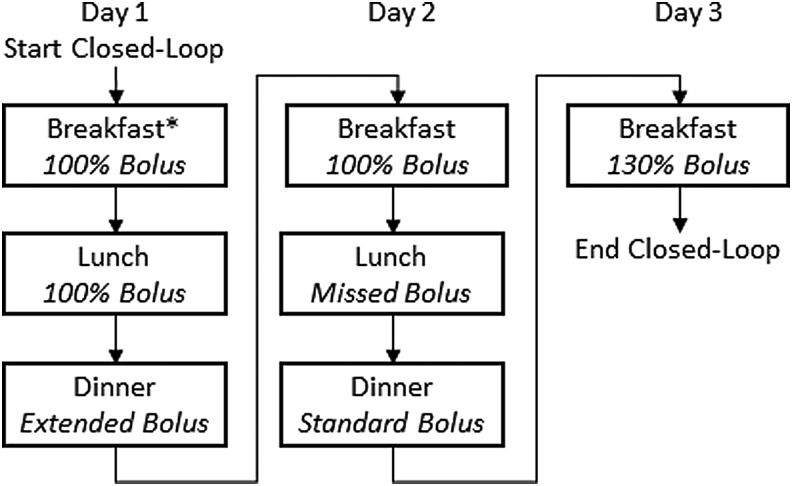
Schematic diagram showing the order of meal scenarios studied for the three HCL study days. Meals were identical for each repeated meal type (breakfast, lunch, and dinner, respectively). *Breakfast on day 1 occurred immediately after HCL start-up and was not included in the meal scenario comparisons. HCL, hybrid closed-loop.

This single-arm multicenter study assessed three specific meal announcement scenarios during a 54-h HCL period in a hotel setting: identical breakfast meals with bolus for 100% or 130% of the estimated CHO amount, identical lunch meals with or without a standard meal bolus, and identical high-fat dinners with standard or extended meal boluses, all occurring on sequential study days in nonrandomized order (Fig. 1). The extended bolus was delivered as 50% of the standard premeal bolus and the remaining 50% delivered for the following 4 h (also known as a dual-wave or biphasic bolus). As a safety constraint, the remainder of the extended bolus was automatically cancelled if the MPC algorithm recommended suspension of insulin delivery based on the insulin on board and glucose trajectory.

The HCL study period was preceded by a 7-day outpatient OL run-in phase, during which subjects managed their diabetes at home per their usual routine using their personal insulin pump and a Dexcom G4 505 Share^®^ AP sensor. Pump settings were adjusted as needed by the investigator, based upon their clinical judgment. The HCL study period began before breakfast on day 1 and ended ∼5 h after breakfast on day 3. Subjects selected meals from a variety of options containing 30–90 g CHO, and >30 g fat for the high-fat dinner, with the identical selected meal repeated for each of the three respective meal types (breakfast, lunch, and dinner). The standard meal bolus amount was calculated based on the CHO content estimated by the subject. A correction or reverse bolus based on a recent fingerstick blood glucose (BG) measurement could be given with the meal bolus at the discretion of the investigator or subject, to account for any difference in the starting BG that may have existed before the comparative meals. The guideline was to consider a correction bolus to a target of 140 mg/dL for a premeal fingerstick BG >140 mg/dL and a reverse correction to the study setpoint of 120 mg/dL for a premeal fingerstick BG <100 mg/dL. No sustained vigorous exercise was performed.

### Study participants

Inclusion criteria for the study were age 18–65 years, type 1 diabetes for ≥1 year, HbA1c value >6% and ≤10% at screening, use of any insulin pump for ≥6 months, and total daily dose (TDD) of insulin ≥0.3 U/kg. Subjects with ≥1 episode of severe hypoglycemia or diabetic ketoacidosis requiring an emergency room visit or hospitalization within the past 6 months, or with hypoglycemia unawareness assessed by the Clarke Questionnaire,^21^ were excluded. Each study site received Institutional Review Board approval and subjects provided written informed consent (Clinicaltrials.gov registration NCT03064906).

### Safety and monitoring

Study staff monitored subjects' status throughout the HCL study period, with hypoglycemia (fingerstick BG <70 mg/dL or symptomatic) or severe hyperglycemia (fingerstick BG ≥300 mg/dL) treated per standard practice.^22^ HCL stopping criteria included unresolved symptomatic hypo- or hyperglycemia, subject request, loss of consciousness or seizure, or BG ≥300 mg/dL and ketones ≥3.0 mmol/L.

### Investigational device

The investigational system used in this study, described previously by Buckingham et al.,^20^ consisted of a modified version of the Omnipod insulin pump (Pod), a modified Personal Diabetes Manager, the Dexcom G4 505 Share AP System, and the personalized MPC algorithm running on a Windows 10 tablet configured with the portable AP system developed at the University of California at Santa Barbara.^23^ Inputs to the algorithm included the subject-specific basal rate profile and TDD of insulin. The algorithm setpoint for this study was 120 mg/dL.

### Outcomes

The primary endpoints were safety parameters of percentage of time the sensor glucose was in a hypoglycemic range defined as <70 mg/dL and hyperglycemic range defined as ≥250 mg/dL during the HCL study period. Secondary endpoints included mean sensor glucose, percentage time with sensor glucose <54, <60, 70–140, 70–180, >180, ≥300 mg/dL, and standard deviation (SD) and coefficient of variation (CV) of sensor glucose values.^24,25^ Outcomes for the three meal announcement scenarios were the 4-h postprandial glucose response to breakfast with 100% versus 130% of the standard meal bolus amount, the 4-h postprandial glucose response to lunch with and without a standard meal bolus, and the 12-h postprandial glucose response to a high-fat dinner with a standard or extended meal bolus.

### Statistical analysis

As the primary endpoint for the study was safety, sample size was not determined by power calculation. Prespecified descriptive statistical analyses were performed for all subjects who entered the study (*n* = 12). Results were summarized for the 54-h HCL study period (overall) and the overnight period defined as 23:00 to 07:00 h. Results were also summarized for the three specific meal announcement scenarios. Outcomes were calculated per subject and summarized as mean ± SD or median (interquartile range, IQR), unless otherwise indicated. Postprandial outcomes for each test case (130% overestimated meal bolus, missed meal bolus, or extended meal bolus) were compared with the standard meal bolus case for the corresponding identical meal using the two-tailed Wilcoxon signed rank test for paired observations, with *P*-values <0.05 considered statistically significant. Statistical analyses were performed using SAS^®^ 9.3 or later (SAS Institute, Cary, NC) and MATLAB (MathWorks, Natick, MA).

## Results

The characteristics of the 12 subjects are reported in Table 1. A summary of the pump setting adjustments for the 7-day OL run-in phase is included in the Supplementary Data (Supplementary Data are available online at http://www.liebertpub.com/dia).

**Table 1.** Characteristics of the Study Population

*Characteristic*	*Subjects (*n* = 12)*
Age, years (range)	35.4 ± 14.1 (20.3–60.6)
Female, %	75
Diabetes duration, years (range)	16.5 ± 9.3 (7.0–37.6)
Insulin pump use duration, years (range)	12.0 ± 8.6 (1.6–28.2)
Insulin dose open-loop, U/(kg·d)^a^	0.58 ± 0.19
Insulin dose HCL, U/(kg·d)^b^	0.56 ± 0.18
HbA1c, %	7.7 ± 0.9

Results are mean ± SD unless otherwise indicated.

^a^ Insulin dose averaged for the 7-day open-loop run-in phase.

^b^ Insulin dose during entire HCL study period.

HCL, hybrid closed-loop; SD, standard deviation.

### Glycemic outcomes

The glycemic outcomes for the 54-h HCL study period overall, during daytime (07:00–23:00 h) and overnight (23:00–7:00 h), are shown in Table 2. The percentage of time with sensor glucose in the hypoglycemic range of <70 mg/dL was median (IQR): 0.0% (0.0–1.2%) during the entire 54-h HCL period and 0.0% (0.0–0.0%) overnight. The percentage of time with sensor glucose in the hyperglycemic range of ≥250 mg/dL was mean ± SD 4.5% ± 3.6% for the overall HCL period and 1.2% ± 4.1% overnight. The percentage of time with sensor glucose in the target range of 70–180 mg/dL was 76.1% ± 8.0% overall and 92.7% ± 13.3% overnight. The mean glucose was 153 ± 15 mg/dL overall and 134 ± 23 mg/dL overnight.

**Table 2.** Glycemic Outcomes During the 54-Hour Hybrid Closed-Loop Period

*Parameter*	*Overall (54-h)*	*Day (07:00–23:00 h)*	*Night (23:00–7:00 h)*
Mean sensor glucose, mg/dL	153 ± 15	162 ± 17	134 ± 23
SD, mg/dL	45 ± 6	48 ± 8	22 ± 12
Coefficient of variation, %	29.6 ± 4.3	29.6 ± 5.2	16.1 ± 7.0
Percentage time in glucose range, %			
<54 mg/dL	0.1 ± 0.2	0.1 ± 0.3	0.0 ± 0.0
	0.0 (0.0–0.0)	0.0 (0.0–0.0)	0.0 (0.0–0.0)
<60 mg/dL	0.1 ± 0.3	0.2 ± 0.4	0.0 ± 0.0
	0.0 (0.0–0.1)	0.0 (0.0–0.1)	0.0 (0.0–0.0)
<70 mg/dL	0.6 ± 0.9	0.8 ± 1.3	0.2 ± 0.6
	0.0 (0.0–1.2)	0.0 (0.0–1.3)	0.0 (0.0–0.0)
70–140 mg/dL	46.4 ± 14.5	36.1 ± 13.6	68.9 ± 29.3
70–180 mg/dL	76.1 ± 8.0	68.6 ± 9.9	92.7 ± 13.3
>180 mg/dL	23.3 ± 8.5	30.6 ± 10.8	7.1 ± 13.4
≥250 mg/dL	4.5 ± 3.6	6.0 ± 4.5	1.2 ± 4.1
≥300 mg/dL	0.9 ± 1.1	1.3 ± 1.6	0.0 ± 0.2

Results are sensor glucose values, mean ± SD or median (IQR) unless otherwise indicated; SI conversion factor to convert glucose to mmol/L, multiply by 0.0555.

IQR, interquartile range; SI, International System of Units.

Additional study information, including a summary of the glycemic measures for the 7-day OL run-in phase (Table S1 and Figure S1), correction boluses given during HCL, and snack consumption unrelated to hypoglycemia, is included in the Supplementary Data.

### Meal challenges

#### Overestimated bolus

**FIG. 2.**
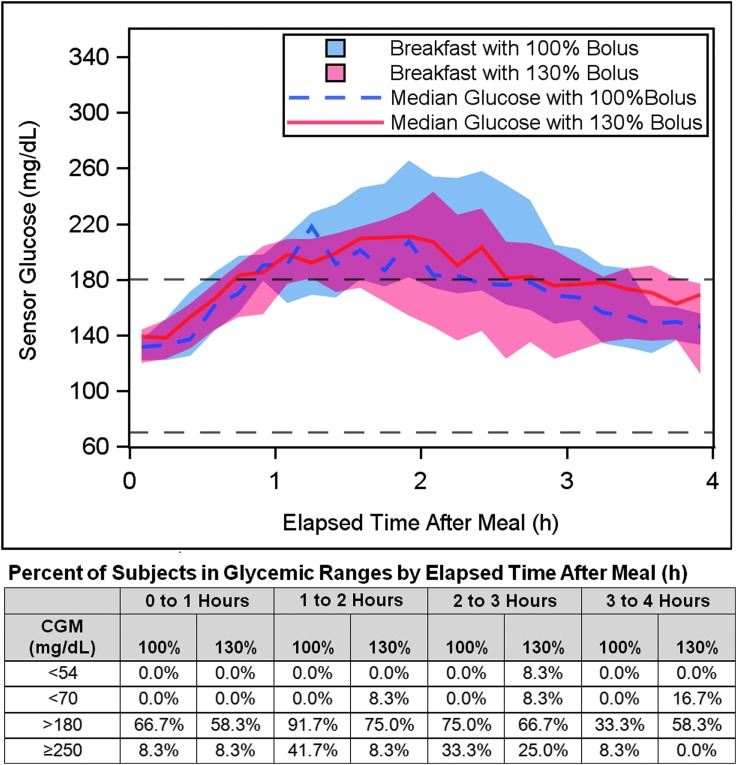
Comparison of glycemic response with a standard 100% meal bolus versus a 130% overestimated bolus for a 4-h postprandial period. Median sensor glucose response is plotted for the 12 subjects for 4 h after a 34–60 g CHO breakfast with standard meal bolus (100% of calculated value, dashed blue line) and overestimated meal bolus (130% of calculated value, solid red line). Each subject consumed an identical meal on the days with 100% and 130% meal bolus. The shaded area represents the IQR. The target range of 70–180 mg/dL is indicated by black dashed lines. The percentage of subjects in various glycemic ranges (hypoglycemia <70 and <54 mg/dL, and hyperglycemia >180 and ≥250 mg/dL) during each hour of the postprandial period is tabulated for each bolus type (100% and 130%) beneath the graph. CHO, carbohydrate; IQR, interquartile range.

The outcomes during the 4-h postprandial period after breakfast with 100% bolus or 130% overestimated bolus are shown in Figure 2 and Table 3. The estimated meal size was 49.4 ± 8.7 g CHO (range 34–60 g). The minimum sensor glucose for the 4-h postprandial period was 121 ± 21 mg/dL for 100% bolus and 114 ± 34 mg/dL for 130% bolus (*P* = 0.69). There was 0.0% (0.0–0.0%) of time <70 mg/dL during the postprandial periods after both the 100% bolus and the 130% bolus (*P* = 0.50), with no subjects spending time <70 mg/dL after the 100% bolus versus two subjects after the 130% bolus (Fig. 2). No subjects consumed supplemental CHO during the postprandial period after the 100% bolus and three subjects consumed supplemental CHO (two with fingerstick BG <70 mg/dL) after the 130% bolus. The percentage time with sensor glucose ≥250 mg/dL in the 4-h period after breakfast was lower with the 130% overestimated bolus (12.5% ± 18.9% and 4.0% ± 9.2% for the 100% and 130% boluses, respectively, corresponding to 30 ± 45 min and 10 ± 22 min, although the difference was not significant [*P* = 0.16]).

**Table 3.** Preprandial and 4-Hour Postprandial Outcomes for Breakfast with Standard 100% Versus 130% Overestimated Meal Bolus

	*Bolus type*		
	*100% Bolus*	*130% Bolus*	P
Glucose values, mg/dL			
Preprandial	126 ± 13^a^	132 ± 13	0.21
Postprandial peak^b^	229 ± 47^a^	219 ± 47	0.35
Excursion^c^	103 ± 49^a^	88 ± 47	0.21
Maximum^d^	242 ± 64	222 ± 44	0.27
Minimum	121 ± 21	114 ± 34	0.69
Mean	182 ± 35	171 ± 34	0.30
AUC,^e^ h · (mg/dL)	230 ± 139	177 ± 97	0.13
	182 (137–318)	172 (101–263)	
Percentage time in glucose range, %			
<54 mg/dL	0.0 ± 0.0	0.4 ± 1.3	1.0
	0.0 (0.0–0.0)	0.0 (0.0–0.0)	
<70 mg/dL	0 ± 0	2.4 ± 5.7	0.50
	0.0 (0.0–0.0)	0.0 (0.0–0.0)	
>180 mg/dL	40.3 ± 28.3	43.5 ± 27.4	0.97
≥250 mg/dL	12.5 ± 18.9	4.0 ± 9.2	0.16
Insulin delivery, U			
Preprandial bolus	5.1 ± 2.1	6.7 ± 2.7	—
Postprandial algorithm delivery	4.3 ± 1.7	3.9 ± 1.7	0.44
Total preprandial+postprandial	9.3 ± 2.3	10.6 ± 3.0	—
Number of subjects consuming supplemental CHO			
Total	0	3	—
With fingerstick BG <70 mg/dL	0	2	—

Results are sensor glucose values, mean ± SD or median (IQR) unless otherwise indicated; SI conversion factor to convert glucose to mmol/L, multiply by 0.0555.

^a^ *N* = 11 due to missing sensor data for one subject.

^b^ Primary peak identified as resulting from meal.

^c^ Postprandial peak minus preprandial glucose.

^d^ Maximum sensor glucose for postprandial period.

^e^ Incremental AUC that is more than the premeal concentration level.

^*^ *P* < 0.05.

AUC, area under the glucose concentration curve; BG, blood glucose; CHO, carbohydrate.

#### Missed bolus

**FIG. 3.**
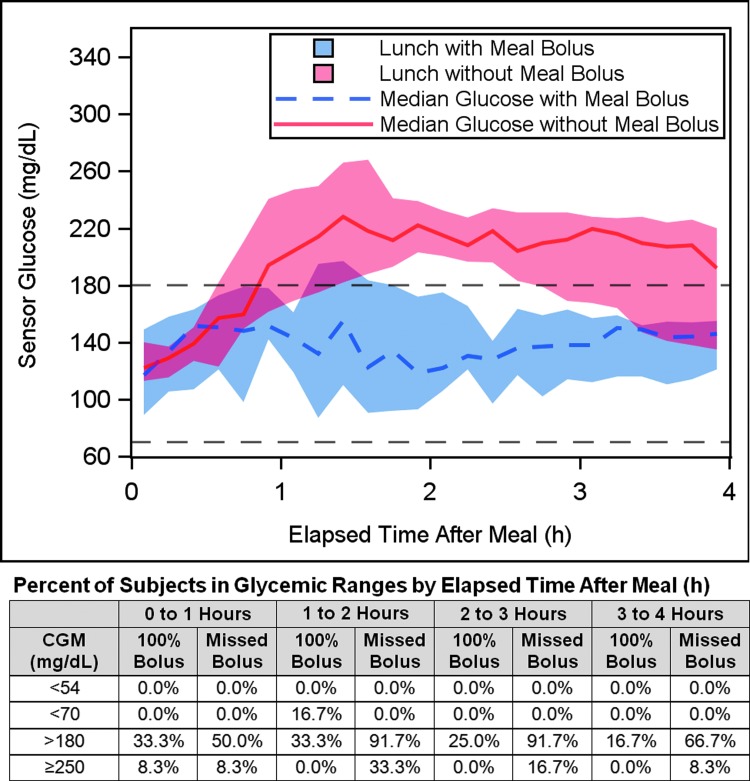
Comparison of glycemic response with a standard 100% meal bolus versus a missed bolus for a 4-h postprandial period. Median sensor glucose response is plotted for the 12 subjects for 4 h after a 30–65 g CHO lunch with (dashed blue line) and without (solid red line) a meal bolus. Each subject consumed an identical meal on the days with and without the meal bolus. The shaded area represents the IQR. The target range of 70–180 mg/dL is indicated by black dashed lines. The percentage of subjects in various glycemic ranges (hypoglycemia <70 and <54 mg/dL, and hyperglycemia >180 and ≥250 mg/dL) during each hour of the postprandial period is tabulated for each bolus type (100% and missed) beneath the graph.

The outcomes during the 4-h postprandial period after lunch with a standard 100% or missed meal bolus are shown in Figure 3 and Table 4. The estimated meal size was 48.1 ± 12.4 g CHO (range 30–65 g). The peak postprandial sensor glucose was 180 ± 45 mg/dL after the 100% bolus and 243 ± 43 mg/dL after the missed bolus (a 35% increase, *P* = 0.001). After the 100% bolus, the six subjects whose sensor glucose rose >180 mg/dL returned less than this threshold within 2.8 ± 2.3 h of the meal. After the missed bolus, the 11 subjects whose sensor glucose rose >180 mg/dL returned less than this threshold within 3.8 ± 1.3 h of the meal. The mean glucose level during the 4-h postprandial period was 141 ± 31 mg/dL after the 100% bolus and 192 ± 32 mg/dL after the missed bolus (*P* = 0.001). There was 0.0% (0.0–0.0%) of time <70 mg/dL during the postprandial periods after lunch with both the 100% bolus and the missed bolus (*P* = 0.5). The percentage time with sensor glucose ≥250 mg/dL in the 4-h period after lunch was lower with the 100% bolus (0.2% ± 0.6% and 10.3% ± 16.5% for the 100% and missed boluses, respectively, corresponding to 0.5 ± 1 min and 25 ± 40 min [*P* = 0.06]). Four subjects consumed supplemental CHO (two with fingerstick BG <70 mg/dL) in the 100% bolus case, versus one subject (no fingerstick BG <70 mg/dL) in the missed bolus case.

**Table 4.** Preprandial and 4-Hour Postprandial Outcomes for Lunch with Standard 100% Versus Missed Meal Bolus

	*Bolus type*		
	*100% Bolus*^a^	*No bolus*	P
Glucose values, mg/dL			
Preprandial	117 ± 41	130 ± 19	0.18
Postprandial peak^b^	180 ± 45	243 ± 43	0.001^*^
Excursion^c^	63 ± 46	114 ± 47	0.01^*^
Maximum^d^	188 ± 37	245 ± 43	0.002^*^
Minimum	98 ± 29	118 ± 18	0.02^*^
Mean	141 ± 31	192 ± 32	0.001^*^
AUC,^e^ h · (mg/dL)	136 ± 99	255 ± 132	0.03^*^
	133 (26–193)	253 (166–362)	
Percentage time in glucose range, %			
<54 mg/dL	0.0 ± 0.0	0.0 ± 0.0	—
	0.0 (0.0–0.0)	0.0 (0.0–0.0)	
<70 mg/dL	1.6 ± 4.0	0 ± 0	0.5
	0.0 (0.0–0.0)	0.0 (0.0–0.0)	
>180 mg/dL	18.4 ± 20.8	59.4 ± 25.2	0.002^*^
≥250 mg/dL	0.2 ± 0.6	10.3 ± 16.5	0.06
Insulin delivery, U			
Preprandial bolus	5.2 ± 2.5	—	—
Postprandial algorithm delivery	3.7 ± 1.9	6.6 ± 2.2	<0.001^*^
Total preprandial+postprandial	8.9 ± 3.4	6.6 ± 2.2	—
Number of subjects consuming supplemental CHO			
Total	4	1	—
With fingerstick BG <70 mg/dL	2	0	—

Results are sensor glucose values, mean ± SD unless otherwise indicated; SI conversion factor to convert glucose to mmol/L, multiply by 0.0555.

^a^ *N* = 11, one subject excluded due to meal size deviation for 100% bolus case.

^b^ Primary peak identified as resulting from meal.

^c^ Postprandial peak minus preprandial glucose, note that this value was negative for two subjects in the 100% bolus case.

^d^ Maximum sensor glucose for postprandial period.

^e^ Incremental AUC that is more than the premeal concentration level.

^*^ *P* < 0.05.

#### Extended bolus

**FIG. 4.**
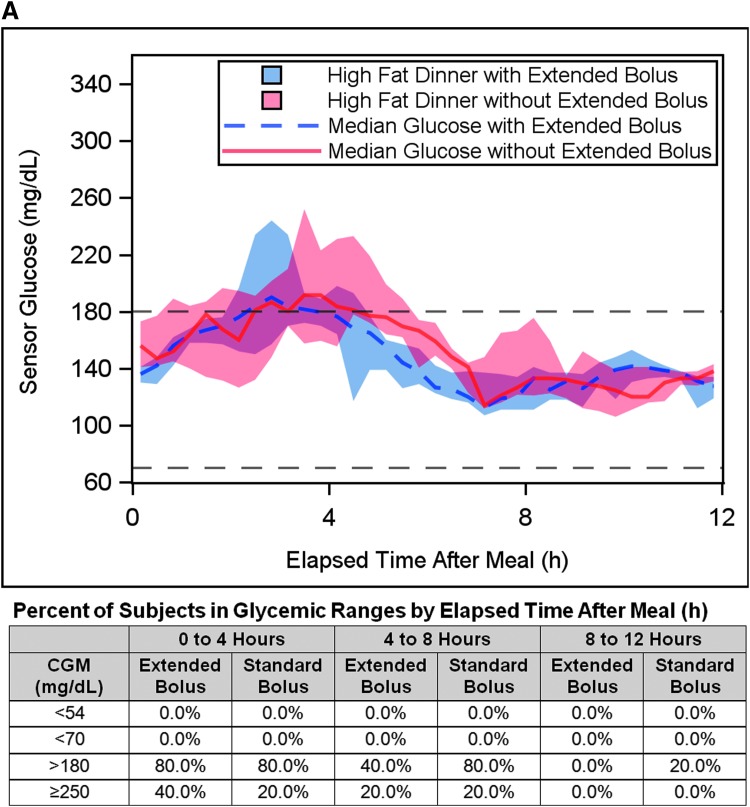

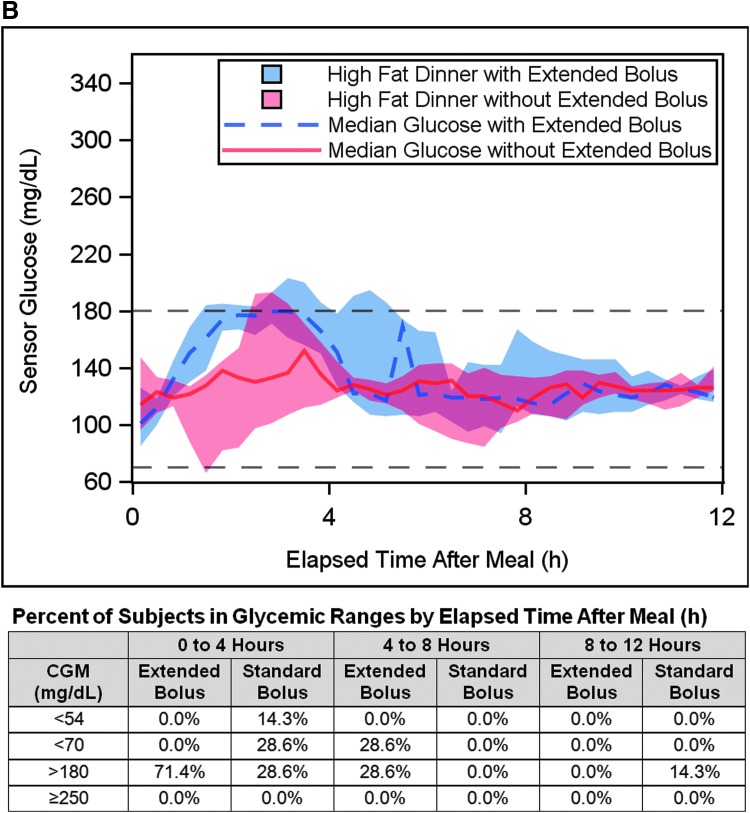
Comparison of glycemic response with a standard 100% meal bolus versus an extended meal bolus for a 12-h postprandial period. Median sensor glucose response is plotted for the 12 subjects for 12 h after a 30–58 g fat dinner with an extended meal bolus (50% bolus upfront and 50% extended for 4 h) (dashed blue line) and without an extended meal bolus (100% bolus upfront) (solid red line). Data are shown separately for **(A)** the five subjects who received the extended bolus for >1 h and **(B)** the seven subjects for whom the extended bolus was canceled by the algorithm within 1 h due to the safety constraint. Each subject consumed an identical meal on the days with the standard and extended bolus. The shaded area represents the IQR. The target range of 70–180 mg/dL is indicated by black dashed lines. The percentage of subjects in various glycemic ranges (hypoglycemia <70 and <54 mg/dL, and hyperglycemia >180 and ≥250 mg/dL) during 4 h segments of the postprandial period is tabulated for each bolus type (extended and standard) beneath each graph.

The outcomes during the 12-h postprandial period after the high-fat dinner with a standard 100% or an extended meal bolus are shown in Figure 4 and Table 5. The estimated meal size was 66.5 ± 20.9 g CHO (range 36–90 g) and 40.3 ± 9.3 g fat (range 30–58 g). The data are stratified by subjects who received the extended bolus for >1 h [*n* = 5, duration of extended bolus median 3.9 h (range 1.8–4 h)] and subjects who had the extended portion of the bolus canceled by the algorithm within 1 h [*n* = 7, duration of extended bolus median 10 min (range 10–20 min)] according to the algorithm safety constraint.

**Table 5.** Preprandial and 12-Hour Postprandial Outcomes for High-Fat Dinner with Standard 100% Versus Extended Meal Bolus

	*Subjects with extended bolus >1 h (*n* = 5)*			*Subjects with extended bolus <1 h (*n* = 7)*		
	*Standard bolus*	*Extended bolus*	P	*Standard bolus*	*Extended bolus*	P
Glucose values, mg/dL						
Preprandial	164 ± 29	134 ± 14	0.06	125 ± 38	106 ± 20	0.38
Postprandial peak^a^	167 ± 24	221 ± 62	0.13	153 ± 42	198 ± 25	0.03^*^
Excursion^b^	2 ± 22	88 ± 51	0.06	28 ± 69	92 ± 38	0.08
Maximum^c^	224 ± 53	228 ± 56	0.81	174 ± 29	201 ± 23	0.08
Minimum	100 ± 20	107 ± 11	0.31	82 ± 23	84 ± 17	0.89
Mean	157 ± 28	153 ± 23	0.81	126 ± 12	139 ± 15	0.02^*^
AUC,^d^ h · (mg/dL)	183 ± 224	305 ± 163	0.31	199 ± 207	434 ± 232	0.11
	114 (2–267)	335 (203–389)		148 (1–427)	542 (255–625)	
Percentage time in glucose range, %						
<54 mg/dL	0.0 ± 0.0	0.0 ± 0.0	—	0.2 ± 0.5	0.0 ± 0.0	1.0
	0.0 (0.0–0.0)	0.0 (0.0–0.0)		0.0 (0.0–0.0)	0.0 (0.0–0.0)	
<70 mg/dL	0 ± 0	0 ± 0	—	1.5 ± 2.7	0.5 ± 1.1	0.75
	0.0 (0.0–0.0)	0.0 (0.0–0.0)		0.0 (0.0–3.5)	0.0 (0.0–0.7)	
>180 mg/dL	23.9 ± 19.4	17.7 ± 18.0	0.31	4.0 ± 6.7	12.7 ± 10.6	0.16
≥250 mg/dL	5.6 ± 12.5	4.4 ± 6.1	1.0	0 ± 0	0 ± 0	—
Insulin delivery, U						
Preprandial bolus	6.7 ± 2.1	3.4 ± 1.0	—	6.1 ± 4.0	3.1 ± 2.0	—
Postprandial algorithm delivery	12.9 ± 2.9	16.1 ± 1.8	0.13	7.7 ± 3.5	9.6 ± 4.5	0.05
Total preprandial+postprandial	19.7 ± 3.4	19.4 ± 1.9	—	13.8 ± 6.3	12.7 ± 5.5	—
Number of subjects consuming supplemental CHO						
Total	0	0	—	3	2	—
With fingerstick BG <70 mg/dL	0	0	—	2	0	—

Results are sensor glucose values, mean ± SD unless otherwise indicated; SI conversion factor to convert glucose to mmol/L, multiply by 0.0555.

^a^ Primary peak identified as resulting from meal.

^b^ Postprandial peak minus preprandial glucose, note that this value was negative for five subjects in the standard bolus case.

^c^ Maximum sensor glucose for postprandial period.

^d^ Incremental AUC that is more than the premeal concentration level.

^*^ *P* < 0.05.

Subjects who received the extended bolus for >1 h (*n* = 5) had a mean preprandial sensor glucose more than the setpoint of 120 mg/dL before receiving the extended bolus (preprandial glucose 164 ± 29 mg/dL for standard bolus and 134 ± 14 mg/dL for extended bolus). The maximum sensor glucose in the 12-h postprandial period after dinner was 224 ± 53 mg/dL with standard bolus and 228 ± 56 mg/dL with extended bolus (a 2% difference, *P* = 0.81). The minimum sensor glucose during the postprandial period was 100 ± 20 mg/dL (range 74–126 mg/dL) with standard bolus and 107 ± 11 mg/dL (range 92–122 mg/dL) with extended bolus (*P* = 0.31). There was no time spent <70 mg/dL during the postprandial period, and a similar percentage of time ≥250 mg/dL: 5.6% ± 12.5% (40 ± 90 min) and 4.4% ± 6.1% (32 ± 44 min) of time for standard and extended boluses, respectively (*P* = 1.0). No subjects consumed supplemental CHO during the postprandial period for either bolus type.

Subjects who had the extended bolus canceled within 1 h (*n* = 7) had a mean preprandial sensor glucose less than the setpoint of 120 mg/dL before receiving the extended bolus (preprandial glucose 125 ± 38 mg/dL for standard bolus and 106 ± 20 mg/dL for extended bolus). The maximum sensor glucose during the 12-h postprandial period after dinner was 174 ± 29 mg/dL with standard bolus and 201 ± 23 mg/dL with extended bolus (a 16% difference, *P* = 0.08). The minimum sensor glucose during the postprandial period was 82 ± 23 mg/dL (range 46–108 mg/dL) with standard bolus and 84 ± 17 mg/dL (range 65–102 mg/dL) with extended bolus (*P* = 0.89). There was no time spent ≥250 mg/dL during the postprandial period, with 0.0% (0.0–3.5%) [0 min (0–25 min)] and 0.0% (0.0–0.7%) [0 min (0–5 min)] of time <70 mg/dL for standard and extended boluses, respectively (*P* = 0.75). Three subjects consumed supplemental CHO (two with fingerstick BG <70 mg/dL) for the standard bolus case, versus two subjects (zero with fingerstick BG <70 mg/dL) for the extended bolus case.

### Safety outcomes

There were no serious adverse events reported, and the HCL period was completed for all 12 subjects with no instances of the stopping criteria being met. In the 648 subject-hours of HCL use, there were 5 hyperglycemic events in 5 subjects involving meter glucose values ≥300 mg/dL, of which 3 required treatment. One of these events resulted in a Pod change due to suspected infusion site failure. There were 9 hypoglycemic events in 5 subjects involving meter glucose values <70 mg/dL, with 10 oral CHO treatments given (10–16 g CHO).

### Percentage time in HCL

The mean percentage of the total HCL study period spent with the system running in closed-loop was 99.2% ± 2.3% (range: 92.2%–100.0%). There was one suspected infusion site failure and one Pod occlusion alarm during the HCL period, each resulting in Pod replacement and correction bolus. The causes for interruption of closed-loop included sensor or Pod replacement, temporary loss of Pod or CGM communication, or loss of system battery charge.

## Discussion

This multicenter feasibility study demonstrated that the Omnipod personalized MPC algorithm performed well and was safe during day and night use for 54 h in adults with type 1 diabetes in a supervised hotel setting. In addition, the robust multiday design with repeated identical meals allowed examination of the MPC algorithm response to a standard 100% bolus compared with the test cases of a 130% overestimated bolus, a missed bolus, or an extended bolus. Repeating identical meals on subsequent days and excluding strenuous physical activity during the HCL period removed potentially confounding factors to isolate the effect of the various meal bolus scenarios. The study design challenged the HCL algorithm to demonstrate appropriate responsiveness to the glycemic trajectory to minimize hypoglycemia in response to an overestimated meal bolus, as well as prevent prolonged hyperglycemia in response to a missed meal bolus, which are both common scenarios expected to occur in real-world use of the device. The feasibility of extended bolus use for high-fat meals during HCL was also evaluated. Overall, the results show that the algorithm was safe in the presence of each of these three meal bolus scenarios.

In the case of an overestimated meal bolus, the primary concern is subsequent hypoglycemia during the postprandial period. In this study, postprandial hypoglycemia <70 mg/dL was avoided for 83% (10/12) of subjects after the 130% overestimated bolus, with a median of 0% of time <70 mg/dL for 4 h and three subjects consuming supplemental CHO (two with fingerstick BG <70 mg/dL). Attenuation of insulin delivery for impending late postprandial hypoglycemia allows reduction of the duration and severity of hypoglycemia that would otherwise be experienced in similar OL scenarios. Our results support the robust performance of the algorithm in cases of overestimated meal boluses. Similar results were reported by Chase et al. examining the response of a single-hormone AP system to a 130% overestimated meal bolus for a 63 g CHO (51–75 g) meal in 40 adults and adolescents with type 1 diabetes: a median of 0% of plasma glucose values ≤70 mg/dL was reported during the 4 h after the overestimated bolus. Two subjects had BG values <70 mg/dL, and there were one hypoglycemic treatment intervention and eight CHO treatments for predicted low glucose during the 4-h postprandial period.^14^

Gingras et al. also reported similar results in a study of a dual-hormone AP system in 20 adult subjects with a meal bolus overestimated by 27% (75 g CHO meal, *n* = 10) or 44% (45 g CHO meal, *n* = 10): median of 0% of time spent <72 mg/dL during the 4 h postprandial period with a total of three episodes of hypoglycemia treated with CHO. Total glucagon delivery (microboluses) was similar in the standard and overestimated bolus cases during the postprandial period, although most of the glucagon was delivered later in the postprandial period with overestimated boluses.^13^ Results of these and the present study show that in response to an overestimated bolus, an AP system may in many cases be able to avoid hypoglycemia occurrence or reduce its severity by attenuating postprandial insulin delivery; however, treatment with fast-acting CHO is still necessary in some cases, even with systems that have automated glucagon delivery.

In the case of a missed meal bolus, the results of this study compared favorably with other recent reports.^14,15^ Chase et al. examined the response of an AP system to a missed meal bolus for a 50 g CHO meal. The present study was able to achieve less postprandial hyperglycemia after a missed bolus, with a median excursion from baseline to peak of 112 mg/dL (mean 114 mg/dL), compared with a median of 156 mg/dL in Chase et al. (mean not reported).^14^ Cherñavvsky et al. studied the effect of a missed bolus for a 30 g CHO snack and underestimated (75%) bolus for an 80 g CHO lunch with a single-hormone AP system in 16 adolescents. The results were 20.7% and 28.2% of time >250 mg/dL during the 4-h postprandial period for the missed snack bolus and reduced lunch bolus, respectively.^15^ The present study demonstrated a lower percentage of time in hyperglycemia ≥250 mg/dL during the postprandial period after a missed meal bolus (10.3%).^15^ The differences in outcomes may be attributed to a number of factors including different control algorithm, meal size, or population. Forlenza et al. included both announced and unannounced meals in their recent closed-loop study of 10 adults and adolescents, with an average of 75 g and 95 g CHO for announced and unannounced meals, respectively.^17^ Similar to the present study, mean CGM was lower for announced than for unannounced meals (141 mg/dL vs. 198 mg/dL, respectively), with 2.2% of time >250 mg/dL after the announced meals versus 23% of time after the unannounced meals.

Owing to the characteristics of currently available insulins, it may be inevitable that increased hyperglycemia will be experienced with a missed bolus; however, an AP system is expected to reduce the severity and duration of hyperglycemia as compared with what would be observed with a missed bolus in OL therapy. Although this study did not include a comparison with OL therapy with missed bolus, Cherñavvsky et al. demonstrated the benefit of an AP by showing that the percentage of time >250 mg/dL during the 4-h postprandial period with AP was reduced twofold from OL therapy under the same meal bolus challenge scenarios (40.3% and 58.5% of time >250 mg/dL for missed and reduced boluses during standard care, respectively).^15^ Such a reduction in hyperglycemia with AP use may improve long-term outcomes for patients who sometimes miss meal boluses.

To our knowledge, this study was the first to evaluate an extended bolus as part of an AP system. An extended bolus gives users the flexibility to deliver a portion of the meal bolus upfront with the remaining portion infused for a set period of time (50% delivered for 4 h in this study), which may be beneficial for high-fat meals that may have delayed gastric emptying,^18^ or for children with unpredictable eating patterns. The purpose of the extended bolus in the OL and HCL setting is the same: to reduce the risk of early hypoglycemia and delayed hyperglycemia that may be encountered with a standard bolus for certain types of meals. Announcing a meal with an extended bolus gives the AP system more information about the anticipated characteristics and insulin requirements of the meal. The use of extended boluses for high-fat meals during HCL in this study was safe. The extended portion of the bolus was canceled in some subjects according to the algorithm safety constraint implemented in this study, which has informed future development of the integration of extended boluses within the personalized MPC algorithm. The ability to use an extended bolus as part of an AP system may provide additional flexibility to users to manage their diabetes according to their preferences and lifestyle.

For overall glycemic outcomes, a review of the literature indicates that HCL systems may be expected to achieve at least 70% of sensor glucose values between 70 and 180 mg/dL with <4% of values <70 mg/dL,^3^ a CV <36%,^26^ and a mean glucose of ≤155 mg/dL, equivalent to an estimated HbA1c of 7.0%.^27,28^ This study exceeded each of these performance metrics, with 76.1% ± 8.0% of sensor glucose values in the target range of 70–180 mg/dL overall. These glycemic control metrics were met in this study even in the presence of overestimated and missed meal boluses that may occur when an AP system is used in a free-living outpatient environment.

The limitations of this study include not having a control arm with a 130% overestimated meal bolus, missed meal bolus, or extended meal bolus in the usual care arm (out of closed-loop). A control arm comparing with standard of care was not included as this was primarily a feasibility study testing safety of the closed-loop system. The study had a relatively short duration of HCL conducted in a supervised hotel setting. Additional challenges to the algorithm may be faced in an unsupervised environment or when the system is used for longer periods of time.

## Conclusions

This feasibility study demonstrated that the Omnipod personalized MPC algorithm performed well and was safe for 54 h of use by adults in the outpatient hotel setting. An AP system with a flexible and responsive control algorithm may be able to minimize glycemic excursions, including both prolonged severe hyperglycemia after a missed or underestimated meal bolus, as well as hypoglycemia after an overestimated meal bolus. In this study, the system was able to maintain good glycemic control within target ranges during the postprandial period in the presence of challenging meal scenarios, including overestimated, missed, and extended meal boluses with high-fat meals. Longer term outpatient studies will assess safety and performance of the algorithm during extended use under free-living conditions in people of all ages with type 1 diabetes.

## Supplementary Material

Supplemental data
