# Supplementary material for: Performance of the Omnipod Personalized Model Predictive Control Algorithm with Meal Bolus Challenges in Adults with Type 1 Diabetes
Source: Diabetes Technol Ther. 2018 Sep 1;20(9):585–95. doi: 10.1089/dia.2018.0138 (PMC6114075; doi:10.1089/dia.2018.0138)
Supplement: Supplemental data [file Supp_Data.pdf]

Supplementary Data

Changes to Pump Parameters During Open-Loop Run-In Phase

In the open-loop run-in phase, nine (75%) subjects had adjustments to their basal rate with increases made for five subjects and decreases made for four subjects. Changes were made to the insulin-to-carbohydrate (CHO) ratios for eight subjects (67%) with increases (decreased insulin delivery) in three subjects and decreases (increased insulin delivery) in five subjects. Correction factors were adjusted for four (33%) subjects with increases (decreased insulin delivery) in two subjects and decreases (increased insulin delivery) in two subjects.

Frequency and Reason for Correction/Reverse Correction Boluses

Correction boluses ($n=6$) and reverse correction boluses ($n=2$) were given with the meal bolus according to protocol guidelines as described in the Study Design section. Correction boluses were also given for high blood glucose (BG) at closed-loop startup ($n=2$), for Pod occlusion/suspected infusion site failure ($n=2$), per protocol for hyperglycemia treatment ($n=3$), and at subject/investigator request ($n=2$).

Supplemental CHO (Snacks)

There were 21 instances of supplemental CHO consumption (snacks without bolus) not associated with hypoglycemia (defined as fingerstick BG <70 mg/dL) distributed among 10 subjects. The contents varied by subject preference and ranged from 1 to 15 g CHO (median 8 g).

Supplementary Table S1. Glycemic Outcomes During the 7-Day Open-Loop Run-In Phase

Parameter	*Overall (7 days)*	*Night (23:00–7:00)*
Mean sensor glucose, mg/dL	153 ± 17	156 ± 28
Standard deviation, mg/dL	54 ± 11	47 ± 13
Coefficient of variation, %	35.0 ± 6.2	30.4 ± 7.3
Percentage time in glucose range, %		
<54 mg/dL	0.8 ± 0.8	0.6 ± 1.3
	0.5 (0.1–1.1)	0.0 (0.0–0.6)
<70 mg/dL	3.5 ± 3.7	2.2 ± 3.5
	2.1 (1.3–4.5)	0.6 (0.0–3.4)
70–180 mg/dL	68.1 ± 10.5	68.4 ± 15.4
>180 mg/dL	28.4 ± 11.3	29.4 ± 17.1
≥250 mg/dL	5.9 ± 5.4	6.0 ± 7.7

Results are sensor glucose values, mean ± standard deviation or median (interquartile range) unless otherwise indicated; SI conversion factor to convert glucose to mmol/L, multiply by 0.0555.

SI, International System of Units.

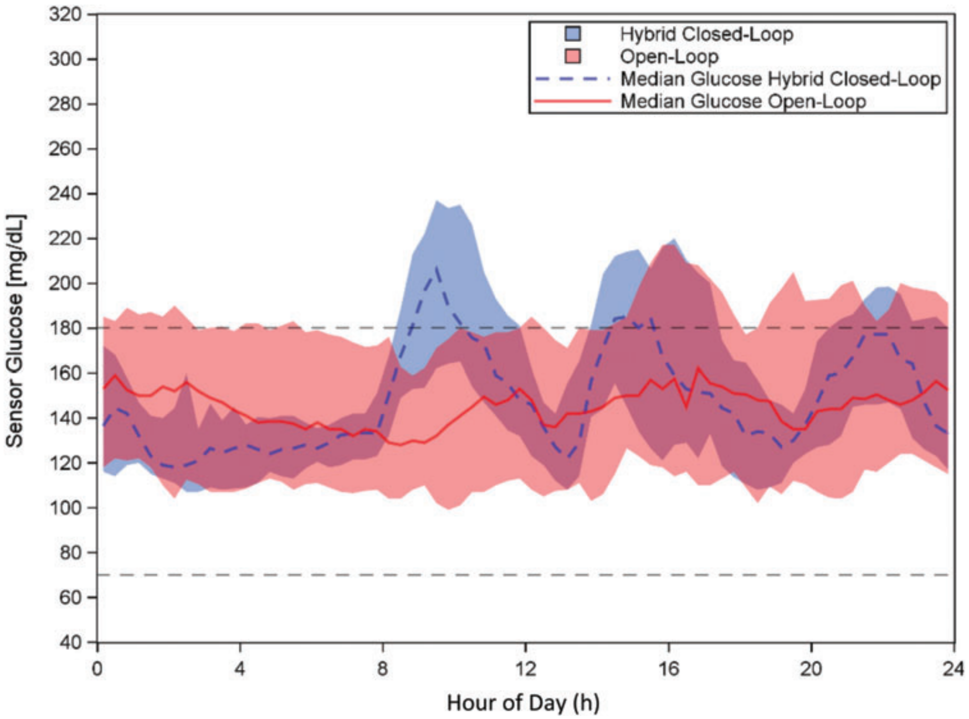

SUPPLEMENTARY FIG. S1. Comparison of 24-h sensor glucose for hybrid closed-loop and open-loop phases. Sensor glucose versus time of day for the 12 subjects during 54 h of HCL control (dashed blue line), with data from 1 week of OL sensor-augmented pump therapy shown as comparison (solid red line). Note that the OL data are from home free-living conditions and, therefore, timing and CHO content of meals may have varied widely, whereas during HCL the subjects ate regular meals of 30–90 g CHO at approximately the same time each day. During HCL, breakfast occurred between 06:18 and 08:57 h, lunch occurred between 12:06 and 13:50 h, and dinner occurred between 18:20 and 19:32 h. The data are presented as median (line) and interquartile range (shaded area) of sensor glucose per time of day across all subjects and days. The target range of 70–180 mg/dL is indicated by black dashed lines. CHO, carbohydrate; HCL, hybrid closed-loop; OL, open-loop.
